# The underlying neural bases of the reversal error while solving algebraic word problems

**DOI:** 10.1038/s41598-022-25442-5

**Published:** 2022-12-15

**Authors:** Noelia Ventura-Campos, Lara Ferrando-Esteve, Irene Epifanio

**Affiliations:** 1 Department of Education and Specific Didactics, Universitat Jaume I, Castellón de La Plana, Spain; 2 Neuropsychology and Functional Neuroimaging Group, Universitat Jaume I, Castellón de La Plana, Spain; 3 Department of Mathematics, Universitat Jaume I, Castellón de La Plana, Spain

**Keywords:** Neuroscience, Mathematics and computing

## Abstract

Problem solving is a core element in mathematical learning. The reversal error in problem solving occurs when students are able to recognize the information in the statement of comparison word problems, but they reverse the relationship between two variables when building the equations. Functional magnetic resonance images were acquired to identify for the first time the neural bases associated with the reversal error. The neuronal bases linked to this error have been used as inputs in 13 classifiers to discriminate between reversal error and non-reversal error groups. We found brain activation in bilateral fronto-parietal areas in the participants who committed reversal errors, and only left fronto-parietal activation in those who did not, suggesting that the reversal error group needed a greater cognitive demand. Instead, the non-reversal error group seems to show that they have developed solid algebraic knowledge. Additionally, the results showed brain activation in the right middle temporal gyrus when comparing the reversal error vs non-reversal error groups. This activation would be associated with the semantic processing which is required to understand the statement and build the equation. Finally, the classifier results show that the brain areas activated could be considered good biomarkers to help us identify competent solvers.

## Introduction

The reversal error was widely described in Clement^1^, where the subjects had to solve tasks such as: “Write an equation using the variables S and P to represent the following statement:"There are six times as many students as professors at this university. "Use S for the number of students and P for the number of professors" (Clement^1^, p. 17). The most common incorrect answer among first-year engineering college students was P = 6 · S. The fact that the variables P and S were presented in opposite positions to those they should occupy led to the name of the error.

The persistence of the error led to the scientific community’s interest in identifying its cause. In these investigations, both quantitative and qualitative experimental designs were used, but the results did not make it possible to conclusively determine the origin of the error.

### Explanatory models for the reversal error

In the study presented in Clement^1^, the difficulty of converting the inequality stated in the statement into an equality is identified as a possible source of the error. In the particular case of the Students-Professor problem, the construction of an equation involves converting a first mental image of the situation, in which there is a ratio of one teacher to six students, into a new image in which the number of teachers is multiplied so that there is a teacher for each student. This set of actions that end with writing the equation is called the hypothetical active operation^1^. The transformation of the initial image into the final one would involve an important effort by the working memory^2^.

In fact, Clement^1^ identifies two possible paths that would lead to reversal error: static comparison (with a semantic origin) and word-order matching (with a syntactic origin). In both cases, the error would be the consequence of a simplification of the hypothetical active operation. According to the static comparison, the explanation assumes that the students reach the mental image in which a teacher and six students are represented, but from this articulation they structure the equation as if it represented an abbreviated explanation of the mental image. Thus 1 professor for every 6 students becomes 1·*P* = 6·*S* (where *P* and *S* act as abbreviations or labels), and from there to *P* = 6·*S* by cancelling the 1. The explanation based on word-order matching would imply that the student does not build a mental image of the described situation, and he/she is limited to performing a translation from left to right [six (6) times as many students (*S*) as ( =) professors (*P*)].

Numerous studies have addressed the question of which of the two models is the usual source of the error. Thus, for example, Rosnick^3^ hypothesized that the static comparison could be the consequence of an incorrect interpretation of the letters, where they would be used as labels that represent measurement units (S would be students instead of number of students). Based on this idea, experimental set-ups were employed where different letters were used to avoid this possible confusion. Thus, Cooper^4^ found that the insertion of a multiplication sign in the equation (e.g., 6·*S* instead of 6S) leads to a decrease in the incidence of the reversal error. However, this study concluded that the use of variables other than the initial letter corresponding to the quantity's name (for example, using *x* to represent the number of students instead of *S*) did not produce variations in the incidence of the error. Fisher^5^ proposed a study in which letters such as Ns were used instead of *S*. The aim of this experiment was to emphasize that the variable represented the number of students and was not just a label to replace 'students'. However, no relationship was found between the use of a type of notation and the greater or lesser incidence of the reversal error. Similarly, in Soneira et al.^6^, no differences were found in the appearance of the reversal error when the students were constrained to using written propositions such as "students" and "number of students" instead of letters.

To assess the explanatory potential of the static comparison, several studies compared the greater or lesser presence of reversal errors depending on whether the contextual clues facilitated the mental construction of a typical situation (for example, in any class it is common for the number of students to be greater than the number of professors). In studies such as those by González-Calero et al.^7^ or Wollman^8^, the results showed that the reversal error rate was not affected by the presence or lack of contextual clues. However, the experimental techniques used did not make it possible to conclude whether this information was really taken into account by the solver.

The experimental set-ups used to analyze the importance of word-order matching in the incidence of the reversal error have been conditioned by the restrictions imposed by natural languages when expressing multiplicative comparisons, at least in English. González-Calero et al.^9^ carried out a comparison of the reversal errors made by Basque-Spanish bilingual students. In this case, the authors tried to take advantage of the fact that, in the Basque language, the order in which the quantities are presented in a multiplicative comparison cannot produce reversal errors due to a literal translation from left to right. The results showed a significant decrease in the reversal error rate when the statements were provided in Basque, which would suggest that the difference in the number of reversal errors would be the effect of applying word-order matching.

Indirectly, Fisher et al.^10^ attribute the lower reversal error occurrence to word-order matching when students are asked to construct the equation using the inverse operation (division in the case of the Students-Professors problem). According to these authors, resorting to linear reading is prevented by forcing the use of the inverse operation. These results were also observed in the investigation by González-Calero et al.^7^.

In short, previous studies seem to show that the origin of the reversal error in different individuals is not necessarily the result of an explanatory model. It is possible that the reversal error is a consequence of inadequate learning.

### Neuroeducation and algebraic reasoning

Functional Magnetic Resonance Imaging (fMRI) studies can help to elucidate the role that specific brain regions play during mathematical processing and development. Dehaene^11^ suggests that intuitive understanding of quantities is associated with activity in the intraparietal sulcus (IPS), included in the parietal cortex. Additionally, the parietal cortex participates in various mathematical tasks, from numerical comparison to more complex processing such as proportions or deductive reasoning^12,13^. De Smedt et al.^14^ also show that different parts of the parietal cortex, such as the bilateral IPS and left angular gyrus, play a crucial role in mental calculation. Moreover, for many researchers, mathematical learning largely involves working memory^15^, which is associated with frontal areas. The working memory has the capacity to store and manipulate information, that is, its function consists of keeping the mental contents active in an accessible state and transforming the contents through mental operations at the same time that it supervises and coordinates the information controlling the operations and mental actions. This makes it possible to perform cognitive tasks that are supported by the maintenance and availability of this information, such as reasoning, understanding, decision-making and problem solving^15^.

Studies on human brain development have increased our knowledge about its maturation and how this maturation could depend on or be important in the learning process. In their study, Gogtay et al.^16^ report a dynamic sequence of grey matter brain maturation that goes from posterior to anterior areas, beginning in parietal areas towards the frontal areas and ending in the temporal cortex. Some cerebral regions, such as the prefrontal areas, seem to mature later^16^, and some authors believe these areas are involved in mathematical cognition and other higher-order processes that develop throughout childhood and adolescence^17^. This could be important in the transition from concrete arithmetic, mostly associated with parietal areas, to the symbolic language of algebra, which involves the fronto-parietal areas. At the age of learning algebra (during adolescence), students need to have developed abstract reasoning skills that allow them to generalize, model, and analyze mathematical equations and theorems^18–21^. Studies such as the one by Qin^18^ show that the brain’s greatest receptivity for learning takes place during adolescence, which makes it conducive to the teaching of algebra. Luna^22^ coincides with this author, stating that, as adults, we would be limited in our ability to "learn", but not in adolescence, even though a brain can experience structural and functional changes due to a learning process at any age^23^.

According to Kieran^24^, the central processes in algebraic problem solving include analyzing the quantitative relationships between the quantities and modelling the structure of these relationships. Several neuroscience studies with adults have examined the processes used for solving word problems and how these processes are acquired. The Lee et al.^19^ study presented a problem-solving task and showed bilateral activation in brain areas of the prefrontal and parietal cortex when the participant transformed the statement into an equation. Additionally, we would like to highlight the importance of understanding the statement of the word problem in achieving a good symbolic translation to algebraic equations. Thus, previous studies^25–27^ show how the fronto-parietal network is also involved in the semantic domain, where the statements of word problems are interpreted for their symbolic translation. However, according to the study by Friedrich et al.^28^, only the prefrontal areas are involved in the syntactic processing of abstract mathematical language (equation). These results agree with the modules proposed by Anderson et al.^20^ during the process of solving algebraic problems: (1) a retrieval module responsible for the recovery of algebraic rules and procedures that had already been learned, associated with the prefrontal cortex; (2) an imaginal module associated with the posterior parietal cortex, which is related to the transformation of language into algebraic equations; (3) finally, a visual module associated with the fusiform gyrus that extracts information about the equation. All these cerebral areas are of great help in analyzing the equations and decoding their information. Therefore, this model is important because it can help us to devise methods to track mental states while solving arithmetic-algebraic problems^21^.

In this study, we present the results of an investigation that uses methodologies from the field of neuroimaging to: (1) describe the brain areas activated when constructing an equation, both correct and incorrect, from a word problem that includes an additive or multiplicative comparison; (2) determine the neural basis of the reversal error; and (3) study classification methods to identify competent solvers using the neuronal data.

## Results

### Behavioral results

The data collected with the app and the fMRI responses are shown in Tables 1 and 2. These tables show the classification of participants into two groups according to their responses, one group that commits reversal error (RE group) and a second group that does not (non-RE group). In both tables, there are two columns, one for the app responses (outside-MR) and the other for responses obtained in the fMRI while performing the RE-task (inside-MR). Table 1 shows the results for the RE group. As the table reveals, there are five participants who commit reversal errors inside-MR, but not outside-MR. The use of fMRI images could impose certain restrictions on task resolution. One of them would be that the participants had a limited time to answer, which might mean that a higher number of participants commit reversal errors than in a pencil and paper environment with no time limit. As explicitly pointed out in studies such as Wollman^8^ or Pawley et al.^29^, the validation of the proposed equation is a step that competent solvers usually undertake and that can cause certain participants to correct the reversal error a posteriori. However, in our research, the restriction of the task resolution time is aligned with the objective of determining which neural processes are involved in the equation construction process when the reversal error is made, and not in post-hoc monitoring processes.

**Table 1:** RE group’s data obtained from App and fMRI responses.

Subject	Outside-MR		Inside-MR	
	RE	Correct	RE	Correct
**1**	**2**	**14**	**19**	**5**
2	12	4	20	4
**3**	**1**	**15**	**18**	**6**
4	11	2	17	7
**5**	**5**	**10**	**20**	**4**
6	15	1	22	2
**7**	**5**	**11**	**22**	**2**
**8**	**3**	**13**	**15**	**9**
9	14	2	18	6
10	12	2	19	5

Bolded participants committed reversal errors inside-MR but not outside-MR. There were 16 items to respond to outside-MR and 24 items inside-MR.

**Table 2:** Non-RE group’s data obtained from App and fMRI responses.

Subject	Outside-MR		Inside-MR	
	RE	Correct	RE	Correct
1	0	16	8	16
2	3	13	6	18
3	0	16	7	17
4	0	16	7	17
5	1	15	6	18
6	0	16	2	22
7	0	16	3	21
8	2	14	7	17
9	0	16	2	22
10	0	16	5	19

There were 16 items to respond to outside-MR and 24 items inside-MR.

Therefore, to analyze the fMRI, we based the classification of the groups on the correct answers on the RE-task obtained inside-MR because the brains of these five participants work like solvers who commit reversal errors when they have limited time and are not given the opportunity to validate the response a posteriori. The app responses helped us to select the participants who committed reversal errors when building the equation and remove those who made other types of errors that were not the objective of this research. Table 2 shows the results for the non-RE group.

Two-sample t-tests conducted on the app’s data revealed no significant differences in RTs between groups (t_18_ = .282; *p* value = .781).

### fMRI results

**Figure 1:**
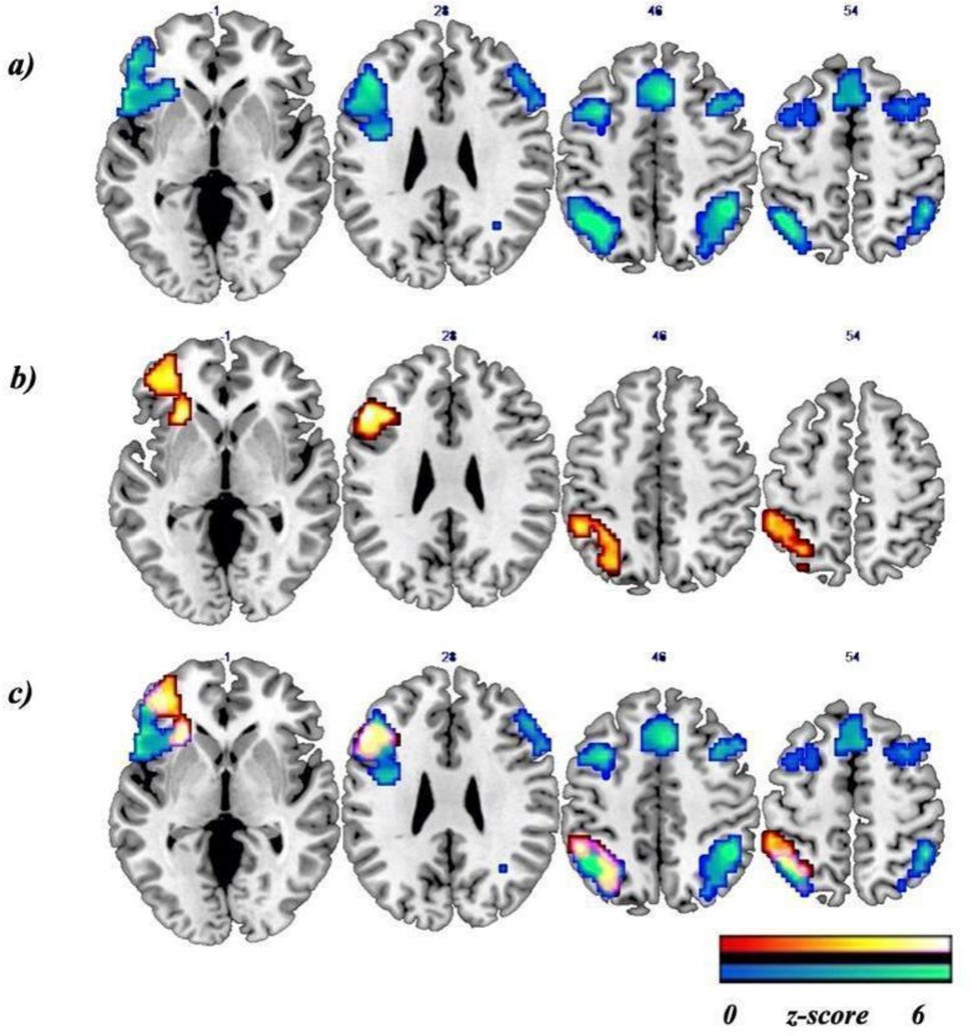
Main effects of the fMRI RE-task. *Note* Results of: (**a**) whole brain one-sample t-test in RE group (blue-green bar); (**b**) whole brain one-sample t-test in non RE group (red–orange bar); (**c**) common regions between RE and non-RE groups (violet-pink). Results were *p* < .05 FWE cluster-corrected using a threshold of *p* < .005 at the uncorrected voxel level and a cluster size of k = 199 voxels and k = 163 voxels, respectively. Coordinates are in the MNI space. The left/right of the image corresponds to the left/right brain hemisphere.

A whole brain one-sample t-test analysis showed significant bilateral activations in frontal and parietal lobes, as well as in the Supplementary Motor Area (SMA)/ cingulate gyrus, in the RE group. For the non-RE group, significant activations were obtained only in the left hemisphere in the frontal lobe extending to the insula and parietal lobe (see Table 3 and Fig. 1 for details).

**Table 3:** List of brain activations as a result of RE-task.

Regions/AAL	BA	cluster	MNI coordinates			z-value
**RE group**						
L Inferior parietal lobule	40	392	− 36	− 55	41	4.46
L Inferior parietal lobule/ Angular			− 39	− 56	40	4.15
L Superior parietal lobule/Intraparietal Sulcus			− 30	− 61	47	3.91
L Superior parietal lobule	7		− 33	− 70	50	3.81
L Inferior frontal gyrus/frontal inferior triangularis	45	1188	− 54	23	5	4.45
L Middle frontal gyrus/frontal inferior triangularis	46		− 45	32	23	4.09
L Inferior frontal gyrus/frontal inferior orbital			− 39	23	− 7	3.84
L Middle frontal gyrus/Frontal middle	10		− 39	47	14	3.79
L Anterior insula			− 27	20	8	3.78
L Precentral gyrus/frontal inferior operculum	44		− 60	17	14	3.64
L Middle frontal gyrus/frontal inferior triangularis	9		− 48	14	29	3.16
R Inferior parietal lobule	40	304	45	− 49	47	4.38
R Angular gyrus			30	− 58	41	3.28
R Superior parietal lobule/ Intraparietal Sulcus			30	− 70	50	3.71
R Medial frontal gyrus/Supplementary motor area		425	6	23	47	3.99
L Superior frontal gyrus	6		− 15	17	68	3.54
R Cingulate gyrus	32		9	23	35	2.73
R Middle Frontal gyrus/frontal inferior operculum	9	294	57	20	35	3.31
R Middle frontal gyrus	8		51	20	44	3.28
R inferior frontal gyrus/ frontal inferior operculum	45		60	20	20	2.83
**Non-RE group**						
L Middle frontal gyrus / frontal inferior triangularis	9	163	− 51	26	26	4.11
L Inferior frontal gyrus/ Anterior insula		184	− 27	26	− 4	3.93
L Middle frontal gyrus/ frontal middle orbital			− 33	47	− 1	3.37
L Inferior frontal gyrus/ frontal middle	10		− 39	47	2	3.15
L Inferior parietal lobule	40	237	− 54	− 43	53	3.57
L Superior parietal lobule/ Intraparietal sulcus			− 30	− 61	47	3.05

L Left, R right, BA Brodmann Area. All the analyses were corrected for multiple comparisons FWE cluster-corrected at *p* < .05.

**Figure 2:**
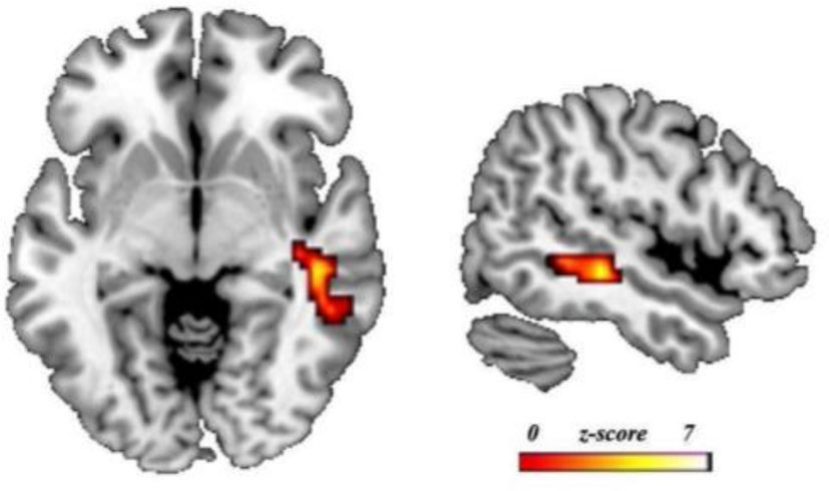
Differences in brain activation in the fMRI RE-task group analysis. *Note* Results of two-sample t-tests between the RE and non-RE groups. Figure represents the RE group > non-RE group contrast. Results were *p* < .05 FWE cluster-corrected using a threshold of *p* < .005 at the uncorrected voxel level and a cluster size of k = 258 voxels. Coordinates are in the MNI space. The left/right of the image corresponds to the left/right brain hemisphere.

The two-sample t-test analysis of the brain differences between groups showed significant brain activation in a single cluster that encompasses the right posterior Middle Temporal Gyrus (pMTG) (x = 51 , y =  − 43 , z =  − 4 , with a Z-value = 3.59) in the RE group compared to the non-RE group (see Fig. 2). The non-RE vs. RE contrast showed no significant activation.

### Classifications results

Table 4 shows the LOU results for the 13 classifiers. The simplest methods yielded better results. The best classifier is the flexible discriminant analysis (FDA), with a correct classification of 16 of the 20 subjects, corresponding to an 80% success rate. Two of the four incorrectly classified subjects appear in shaded rows in Table 1. The second and third best classifiers are the linear discriminant analysis (LDA) and logistic regression (LR), both with a 70% success rate, classifying 14 of the 20 subjects correctly. Both methods classify the same six subjects incorrectly. Three of them appear in shaded rows in Table 1. Note that the subjects who were classified incorrectly with FDA were also classified incorrectly with LDA and LR.

**Table 4:** LOU results for the 13 classifiers.

Method	Accuracy	Proportion
FDA	16	0.8
LDA	14	0.7
LR (variable selection)	14	0.7
NN (Projection Pursuit)	12	0.6
LDA with stepclass	12	0.6
RDA	11	0.55
PLDA	10	0.5
RF	9	0.45
1-KN	9	0.45
QDA	8	0.4
SVM (default parameters)	8	0.4
PDA^30^	8	0.4
SVM (adjusted parameters)	7	0.35

The Accuracy column contains the number of correct predictions, whereas the Proportion column contains the success rate.

## Discussion

The main goals of this study were, first, to investigate the underlying neuronal bases associated with the reversal error phenomenon and, second, find the optimal classification method for these neuronal data to determine whether the activity of the brain areas associated with the two types of solvers could predict the reversal error. Therefore, we aimed to find out whether these areas were able to identify competent solvers. To accomplish these objectives, we separated participants who performed an algebraic problem-solving task with an associated reversal error into two groups (RE group and non-RE group), depending on their responses given inside-MR. To achieve the first objective, we analyzed the fMRI of participants who performed the RE-task in order to obtain the main brain effects derived from committing the reversal error. Two main results were obtained. First, the neural activation elicited by the RE-task differed in the two groups when they processed the task. The RE group showed brain activation in the bilateral inferior and middle frontal gyrus, superior and inferior parietal gyrus, including the IPS and angular gyrus, left insula, and SMA/cingulate gyrus. The non-RE group showed activation only in the left hemisphere, in the inferior and middle frontal gyrus, inferior and superior parietal gyrus, including the IPS, and the insula. Second, to identify differences between groups in specific problem-solving processes when a reversal error is committed, we compared the RE group and the non-RE group. We found activation in the right pMTG.

As expected, both groups obtained greater activation in frontal and parietal areas. These regions have previously been associated with processes that have higher working memory or attentional demands^31,32^ and with quantitative processing^33^, which occurs in processes involving symbolic algebra. With regard to the role of parietal and frontal regions in algebra problem solving, the results of Danker and Anderson^34^ confirm their involvement, but they also highlight how tightly they are intertwined. Although the parietal areas have greater activation in imaginal operations, and frontal areas have greater activation in retrieval operations, both regions were involved in both the transformation and retrieval stages of the problem-solving task^34^.

Regarding the frontal activations, as Owen et al.^32^ also reported, on the one hand, in our study, the middle frontal gyrus (BA 9/46) was found to be involved in monitoring and manipulation within working memory, response selection, and implementation of strategies to facilitate memory. Therefore, it played an essential role in increasing task performance, organization of material before encoding, and verification and evaluation of representations retrieved from long-term memory^35–42^. On the other hand, the inferior frontal gyrus (BA 45) has been specifically implicated in a similarly diverse but distinct set of cognitive processes, including the selection, comparison, and judgment of stimuli held in short-term and long-term memory, holding non-spatial information on-line, stimulus selection, and the elaborative encoding of information into episodic memory^36,43–47^. Thus, evidence seems to indicate that the bilateral frontal areas are highly involved in processes implicated in transforming information embedded in word problems into equations. Furthermore, left frontal activation is frequently associated with verbal working memory tasks^31^. Therefore, this association and the behavioral findings of Lee et al.^48^, who show that verbal working memory tasks predict better algebraic word problem performance, would explain the left lateralized activation in the non-RE group while performing the RE-task.

Activation in the parietal cortex has been shown in various mathematical tasks ranging from numerical comparison to more complex processing, such as proportions or deductive reasoning^12,13^. It is also involved in the implementation of stimulus response mapping^49–54^ and the storage of working memory contents^55^, as well as executive manipulation of acquired facts^56^. Furthermore, the IPS results were expected because this brain area is associated with quantitative processing^33^ on numerosity habituation tasks^57^, mental arithmetic tasks involving symbolic versus non-symbolic conditions^58^, and magnitude comparison processing^59^, the process used by participants to help solve the RE-task. Activation of the IPS has also been found on algebraic problem-solving tasks that show the brain differences between algebraic equations and mental number line conditions^60^. Terao et al.^60^ found that the bilateral IPS was activated in the mental number line condition, whereas the algebra equation condition activated the IPS, but largely left lateralized. Regarding our results, the finding by Terao et al.^60^ has great relevance in our study because only the competent solvers, the non-RE group, who selected the correct equation (which coincided with the equation they had previously created in their head), activated the left IPS. Moreover, the Terao et al.^60^ findings also reveal that the IPS is sensitive to the condition on mathematical tasks. Thus, in our results, we can rule out, as in Lee et al.^19^, that the IPS activation merely reflects exposure to numbers, given that similar numeric stimuli were presented in the experimental and control conditions. We found IPS activation when we subtracted the control condition from the experimental condition (experimental > control contrast).

On the one hand, the cingulate gyrus (BA6/32) has been related to error detection^61,62^ and integration of information^63^, and it is often involved in increased effort, complexity, or attention^64,65^. Arsalidou and Taylor^66^ suggested that the cingulate gyri implement cognitive goals by integrating available information. On the other hand, the insula is associated with error processing^67^ and execution of responses^68^. Thus, the cingulate gyrus and the insula can work together in initiating attentional control signals and detecting important stimuli^69^. Both areas of the brain were bilaterally activated in the RE group. Consequently, we can assume that this group requires high attentional processing in order to carry out the task. However, the non-RE group only activated the left insula, which we interpret as the processing (or control) of the error for the execution of the response.

The results for the RE group during the RE-task overlap with the bilateral fronto-parietal network, with the cingulate gyrus and anterior insula, found in the study by Lee et al.^19^ (see Table 2) using the two methods studied: the model method (Singapore method) and the symbolic method. Their research studied the similarities and differences in the cognitive processes for representing algebraic word problems with these two methods. On the task, the experimental condition required more extensive magnitude comparison (only additive) and working memory engagement than the control condition, as in our RE-task. It should be noted that in the Lee et al.^19^ task, the reversal error is associated in the statement, but the solutions participants propose in choosing the answers facilitate decision making, which keeps them from making this type of error with the symbolic method. For example, when they say “fewer than”, they are expected to choose the subtraction option, which is the correct one, instead of the addition option (see Fig. 2^19^). They are not given the option that the correct answer could involve addition (using algebraic transformation) or subtraction with the variables presented in opposite positions, which would allow them to commit a reversal error. Therefore, the final results include both types of participants, those who make reversal errors and those who do not. For this reason, the RE group results are very similar to those obtained by Lee et al.^19^ because in the fMRI, the main effects are the combination of all the significant group activations. This finding gives greater reliability to our results and reveals the network associated with solving complex algebra word problems.

A few neuroimaging studies have examined algebraic word problems (e.g.^18–21^), and our findings are consistent with the areas involved in these problem-solving tasks. However, no fMRI studies have examined the phenomenon of the reversal error. In relation to differences between groups found in the main effects of the RE-task, our results revealed that the non-RE group activated the fronto-parietal network only in the left hemisphere, showing that fewer areas are needed to perform the RE-task. In previous studies on learning/training, lower activation in areas associated with the task after learning has often been interpreted as an indication of better neural efficiency, which may allow participants to respond by making fewer mistakes^70^. Moreover, this effect of decreased activation is typically observed after training on higher cognitive tasks, which could be the case of our RE-task, and lower activation is associated with increased neural efficiency, which means that fewer neurons are needed to give a fast and accurate answer to the task^71^. Although our research is not a longitudinal study where learning or training has been carried out, we understand that a formal algebra learning process has taken place during elementary school, high school, and university. Some subjects perform problem-solving without committing reversal errors and, therefore, are competent solvers, and others, although having received similar teaching–learning, make reversal errors when performing the task. This seems to reinforce our hypothesis that it is possible that the reversal error is a manifestation of a brain function resulting from inadequate learning.

The comparison of the RE and non-RE groups showed activation in the right pMTG in the RE group, compared to the non-RE group, associated with semantic processing and inhibition processes needed to form non-trivial associations. Although the left hemisphere is dominant in language processing, the right temporal cortex processes the novel semantic information and novel meaning of idioms^72–74^ and the right pMTG plays a crucial role in verbal insight problem solving^75–79^. More specifically, Shen et al.^80^ point out that “the right pMTG may undergo sustained activation for weak meanings of knowledge nodes or distant conceptual associations that are essential for insight” (p. 363). Parsons and Osherson^81^ conclude that the right pMTG is used when subjects solve problems by using deductive reasoning and in the review conducted. Therefore, this result seems to show that the right pMTG is involved in processing new semantic information, possibly because the situations presented are not familiar, and the subject has to decode them in order to give them meaning in the translation into algebraic language. It should be noted that, although in the control condition the effect of syntactic translation was eliminated, this does not mean that the activation obtained is the result of performing only the static comparison model. Moreover, based on these results, we cannot know which model is the predominant one in making the error, given that it could be both, but it does show us that the right pMTG has a significant and powerful activation (and this only occurs if the majority of the participants activate this area). Therefore, we can suggest that the RE group makes an effort to understand the statement and translate its meaning into the equation in either of the two models. Moreover, the use of one model or the other seems to be the only heuristic used to solve the proposed problem. This leads us to investigate the neural differences in the RE group in each model because it seems that they have a semantic basis in common.

Regarding the goal of classification, after testing with 13 classifiers, the methods that best classify in our case, taking into account that the sample is small, would be the FDA, followed by the LDA and LR, which have the same percentage of success. These are simple methods, and our results confirm Hand’s^82^ suggestion that the simplest classical methods often work better than more recent and sophisticated methods, due to uncertainties and arbitrariness, and that this could be especially true with real problems. Furthermore, this classification is capable of detecting participants who made a reversal error inside-MR but did not do so in a "pen and paper" environment with unlimited time (outside-MR). Hence, this is another future research challenge we propose: what is the explanation for this type of student?

Finally, these results contribute valuable information for continuing with this educational neuroscience research by using these areas in a functional connectivity study and finding the brain network of competent solvers.

## Conclusion

Our findings show that, on the one hand, the RE group needed more resources and a greater cognitive demand, and so there was greater activation of the bilateral network associated with working memory, attention processes, and executive function. Furthermore, in this group, the right pMTG was required to understand the statement and translate its meaning into the construction of the equation. On the other hand, the non-RE group showed activation in the left lateralized areas, associated with greater activation during a verbal working memory task involved in better algebraic word-problem performance and symbolic processing in the algebra problem-solving condition. This result seems to show that these participants are competent solvers and have developed solid algebraic knowledge, in terms of the meaning of the variable and the logic of the process of constructing an equation. In short, these brain areas seem to be the ones that are linked to competence in solving algebraic problems. Moreover, the results show that the brain areas activated and introduced as classification variables could be considered good biomarkers to help to identify competent solvers.

To our knowledge, this is the first study to investigate underlying neural bases in the commission of reversal errors during algebraic word problem solving and the key areas of algebraic competence.

## Methods

### Participants

In this study, participants were 37 students at the *University Jaume I* with ages ranging from 18 to 26 years. Finally, only 20 healthy right-handed (10 women) adults with ages ranging between 18 and 25 years participated in this study. These participants were divided into two groups: the *non-RE group* (10 subjects, 5 female; mean age: 21.7, SD: 2.95), who responded correctly to more than 50% of the options on the task; and the *RE group* (10 subjects, 5 female, mean age: 21.3, SD: 2.36), who failed more than 50% of the options on the task and, therefore, made reversal error. The other 17 participants were excluded because: 11 had excessive head movement in the MRI, and six seemed to respond randomly inside the MR. All participants received remuneration for completing the study. The Ethical Committee of Universitat Jaume I approved the research project and this study was performed in accordance with relevant guidelines and regulations. All participants gave informed written consent prior to participation.

All participants were assessed with the Matrix Reasoning subtest (Wechsler Adult Intelligence Scale version III-R) to assess their intelligence quotient (IQ) (RE-group: mean = 11 ± 3.09; non-RE group: mean = 12.30 ± 2.83) and the Digit Span subtest to measure working memory (RE-group: mean = 16.70 ± 3.12; non-RE group: mean = 15.67 ± 3.04). Between-group differences (two-sample t-test) in gender distribution, age, IQ, and working memory were non-significant.

The study exclusion criteria were the presence of neurological and medical illness, trauma with loss of consciousness lasting more than one hour, and the typical resonance exclusion criteria such as iron prostheses and dental implants.

### Experimental paradigm

Participants completed the problem-solving task with the associated reversal error (RE-task), based on the experimental procedure by Lee et al.^19^ This task was adapted to an fMRI block design. Visual stimuli were presented electronically using E-Prime software (Psychology Software Tools, Pittsburgh, PA), professional version 2.0, installed in a Hewlett-Packard portable workstation (screen-resolution 800 × 600, refresh rate of 60 Hz). Participants watched the laptop screen through MRI-compatible goggles (VisuaStim, Resonance Technology, Inc., Northridge, CA, USA), and their responses were collected via MRI-compatible response-grips (NordicNeuroLab, Bergen, Norway). The E-Prime’s logfile saved each participant's accuracy and reaction time (RTs) to each stimulus.

#### RE-task

**Figure 3:**
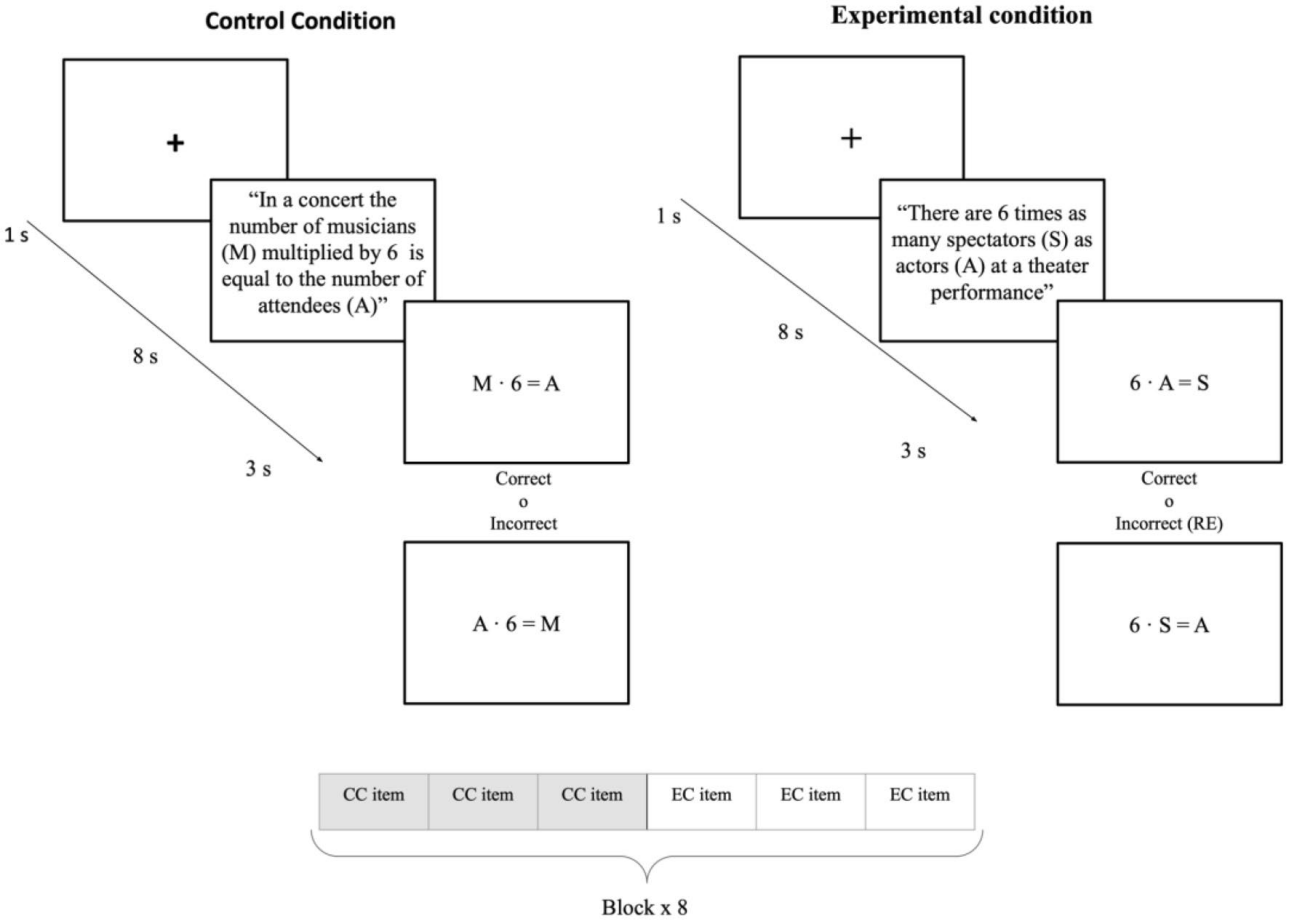
Schematic representation of the experimental design of the RE-task in fMRI. *Note CC* Control Condition, *EC* Experimental Condition. Each block presented with the same item corresponds to one of the eight experimental statement combinations (Multiplicative-Increased-Contextual, Multiplicative-Increased-Non Contextual, Additive-Decreased-Contextual, etc.).

The task consisted of two conditions (control and experimental). In all, participants completed 48 trials (24 control and 24 experimental), divided into 8 blocks, one for each type of statement (item). Within each block, participants were presented with the experimental and control conditions (three different statements and responses per condition in each block). The sequence of the blocks was randomized and balanced for difficulty. On each trial, there was a 1 s fixation point, 8 s when the statement of the word problem in Spanish was presented, and 3 s for the equation response, which was correct 50% of the time. Participants had to respond by indicating whether the equation was correct or incorrect depending on the statement previously presented. The incorrect equation was the reversal error. Participants had to give manual responses with their thumb using a response-grip button. To remove brain activation due to the motor response effect from always pressing the button with the same hand for correct and incorrect answers, 50% of the subjects had to give their responses with the right (left) hand if the answer was correct (incorrect), and the other 50% had to respond with the right (left) hand if the answer was incorrect (correct). The entire task lasted 9 min 36 s. The block organization and timing details are presented in Fig. 3.

The experimental statements used consisted of eight items (2 × 2 × 2) with discrete quantities: (1) multiplicative or additive; (2) increasing (times more than, more than) or decreasing (times less than, less than) comparisons [in Spanish, the multiplicative comparison “X times as many as” is typically constructed by using “X veces más que” (with a literal translation “X times more than”) and “X veces menos que” (with a literal translation “X times less than”)]; and (3) the presence or not of contextual clues that make it possible to determine what quantity is typically greater. The control sentences used the same structure as the experimental condition: eight items with discrete quantities, where the statement does not involve a reversal error because the translation of the statement to the equation is direct (for example, in an academy, the number of tutors (T) plus 9 equals the number of students (A) solution: T + 9 = A).

The design of the control condition for the RE-task has two purposes. On the one hand, the subtraction method^83^ is one of the techniques used in neuroimaging based on comparing fMRI signals between two conditions (experimental > control). Thus, the brain activation obtained when the subject is performing the control condition is subtracted from the brain activation acquired when the subject is performing the experimental condition. In this way, brain activations associated with the simple process of verbal problem solving are eliminated, that is, the brain areas associated with reading the statement and translating from sentence to equation, as well as the occipital areas related to a visual task. Thus, only brain activations associated with the process of solving the problem with the reversal error in the statement were obtained. On the other hand, the control condition was designed so that the equation is a syntactic (lineal) translation of the statement, in order to eliminate the effect of solving the problem in this way.

Participants received oral instructions about how to do the task, and they performed a 5 min practice task before performing the RE-task. On the practice task, participants performed four blocks exclusively with the control condition, in order to become familiar with the stimuli presentation and the response buttons. A similar laptop with the same display features and the same hardware for manual responses was used outside of the scanner. Participants were asked to answer as accurately as possible.

### Behavioral task

In order to make a better selection of the groups based on the reversal error, we carried out a behavioral data collection based on a computer application similar to the one used in González-Calero et al.^7^, where the participants had to build a mathematical equation for each of the statements presented to them. The responses collected from the app were classified as correct answers, reversal errors, and other errors, eliminating from the sample the participants who made errors in translating the equation that were not associated with the reversal error (i.e., other errors). This process was carried out after the fMRI session.

**Figure 4:**
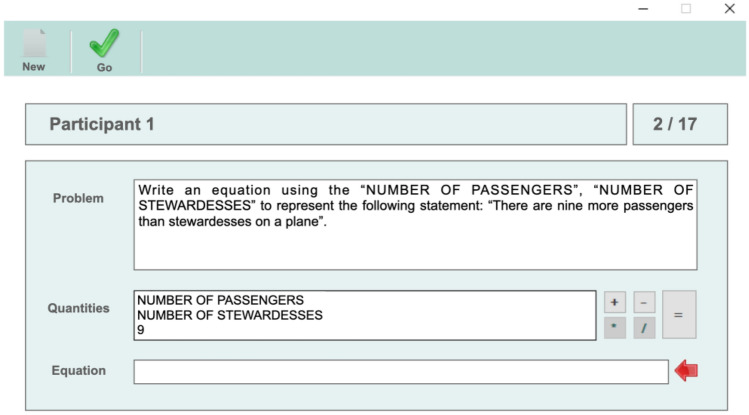
Application implemented for data collection in studies on the reversal error^7^. *Note* By clicking on each of the variables (*quantities*) and operation signs (+, −, *, / and =; right to *quantities*), participants had to write the equation that corresponded to the statement of the problem. The rectangle called *Equation* contains the variables they clicked. After that, they validated their equation by the *Go* button.

The task contained a total of 16 statements (2 × 2 × 2 × 2) in Spanish, focusing on: (1) whether the comparisons are multiplicative or additive; (2) whether the comparisons are increasing (times more than, more than) or decreasing (times less than, less than); (3) contextual or non-contextual clues; and (4) discrete or continuous amounts. Thus, subjects could use only multiplication/division or addition/subtraction to express the equation. To construct the equation, we made it easier by giving them the variables and quantities they had to use (see Fig. 4). It should be noted that the response time was unlimited. The app’s logfile saved the equations made by each participant and their RTs.

### Neuroimaging data acquisition

Functional MRI data were acquired using a 3 T Philips Achiva scanner. For task-fMRI, a gradient-echo T2*-weighted echo-planar image (EPI) MR sequence covering the entire brain was used (TR/TE = 3000/30 ms, matrix = 80 × 80 × 40, flip angle = 90°, voxel size = 3 × 3 × 3.5). A total of 200 volumes were recorded. Before the fMRI sequences, a high-resolution structural T1-weighted MPRAGE sequence was acquired (TR = 8.4 ms, TE = 3.8 ms, matrix size = 320 × 320 × 250, voxel size = .75 × .5 × .8 mm). All the scanner acquisitions were performed in parallel to the anterior commissure-posterior commissure plane (AC-PC), and they covered the entire brain. Participants were placed in a supine position in the MRI scanner. Their heads were immobilized with cushions to reduce motion degradation, and they were asked to minimize their head movement.

### Behavioral analysis

The responses collected from the app were classified as *correct, reversal error,* and *other errors.* In the case of multiplicative comparison (using the example in Fig. 2), if we called P (number of passengers) and S (number of stewardesses) the compared quantities, an equation without a reversal error would be P = 9·S, and so any answer that could be reduced to this equation using correct algebraic transformations would be considered correct (e.g., S = P/9). In a similar way, an equation that could be reduced to S = P·9 would be classified as a reversal error. Any other equation would be considered another type of error, and the participant who made more than 25% of other errors would be excluded from the sample. In the case of additive comparisons, an analogous criterion was used.

In terms of RTs, participants' performance was processed with the IBM SPSS Statistics software (Version 22 Armonk, New York, USA). A two-sample t-test was conducted to show the differences in RTs between groups in building the equation using the app.

### fMRI analysis

#### Preprocessing

Preprocessing and statistical analyses of fMRI data were conducted with SPM12 (Wellcome Trust Centre for Neuroimaging, London, UK), supported by the MatLab software language. Prior to preprocessing, each subject’s fMRI data were aligned to the AC-PC plane by using his/her anatomical image. Then, standard preprocessing was conducted, which included head motion correction, where the functional images were realigned and resliced to fit the mean functional image. No participant had a head motion of more than 2 mm maximum displacement in any direction or 2° of any angular motion throughout the scan. Afterwards, the anatomical image (T1-weighted) was co-registered to the mean functional image, and the transformed anatomical image was then re-segmented. The functional images were spatially normalized to the MNI (Montreal Neurological Institute, Montreal, Canada) space with 3 mm^3^ resolution and spatially smoothed with an isotropic Gaussian kernel of 8 mm Full-Width at Half-Maximum (FWHM).

#### Statistical analysis

The experimental effects in each voxel were estimated in the context of the General Linear Model^84^.In the first-level analysis, we modeled the conditions of interest corresponding to the RE-task (contrast image: experimental > control). The blood- oxygenation level-dependent (BOLD) signal was estimated through the convolution of the stimuli with the canonical hemodynamic response function (HRF). Six motion realignment parameters were included to explain signal variations due to head motion, that is, as covariates of no interest.

The contrast images resulting from the first-level analyses were used for statistical inference in the second-level analyses. In the second-level analyses, a whole-brain one-sample t-test was conducted in order to study the brain regions involved in the main RE-task effects for each group. Next, a two-sample t-test was performed to compare groups at the task level. The statistical criterion was set at *p* < 0.05, using Family-wise error (FWE) cluster-corrected for multiple comparisons (voxel-level uncorrected threshold of *p* < 0.005 with a critical cluster size). According to Friston et al.^85^ the cluster-level inferences are generally more powerful than voxel-level inferences.

We performed a sensitivity power analysis with the G*Power software^86^, using a power of 0.8 and an α error of 0.05 for the group analysis. The required effect size, given our sample size, equals 0.85, suggesting an adequate sensitivity to large effects^87^. Note that this is a "generic" power analysis involving high-dimensional data. Large effects are frequently at issue in fields such as experimental and physiological psychology, fields that are characterized by the study of potent variables or the presence of good experimental control or both^87^.

### Classification analysis

Classification into the RE and non-RE groups or classes by leave-one-out cross-validation was carried out with the R program (https://www.r-project.org/), where the eight clusters activated brain areas, as the regions of interest (ROI) that reflect the brain areas associated with performing the problem-solving task with reversal error *(i.e., RE-task)*, were used as input variables (see Table 3). Then, we selected the mean time course of all the voxels of each ROI to determine the variables. Because the database was small, it was better to use simple models to avoid overfitting. Therefore, we used simple classifiers such as LDA or LR because they would surely yield better results than more sophisticated classifiers. Nevertheless, we also applied a large number of classifiers and evaluated their predictive capacity, particularly accuracy.

As^82^ explains, the simplest classical methods can often work better than more recent and sophisticated methods due to uncertainties and arbitrariness. Note that in our problem we considered a threshold for defining the classes. Moreover, different types of people (making more or fewer reversal errors) can be found within the same class. However, the output variable was finally reduced to two groups.

To estimate performance, we tested 13 classification methods, using leave-one-out cross-validation (one subject was left out each time, the model was adjusted without this data, and finally the prediction was made for that subject). Therefore, 20 (the sample size) different models were estimated for each of these methods: LDA (with and without selection of variables), quadratic discriminant analysis (QDA), FDA, regularized discriminant analysis (RDA), penalized classification using Fisher’s linear discriminant (PLDA), penalized discriminant analysis (PDA), LR, neural networks (NN), support vector machine (with default parameters and cross-validation estimated parameters) (SVM), random forests (RF), and K-neighbors (KN)^88^.

### Limitations and future directions

This study has a few limitations. The main one is the small sample size. We found that we had to eliminate almost half of the sample due to head movement problems in the MRI and poor performance of some participants. Due to the high economic cost of this type of study and the high demand in hospitals, it was impossible for us to increase the sample size. Even so, this study provides the first results on the neural bases underlying the reversal error. Future studies should replicate these results in larger samples.

Moreover, the results open up new research questions, such as: What happens to students who make a reversal error inside-MR but not in the "pen and paper" environment with unlimited time (outside-MR)? Could a longitudinal study utilizing a learning process help to predict the key areas associated with algebraic learning? In addition, it would be interesting to study functional connectivity in a learning process or understand the neural correlates of error detection.

## Data Availability

The MRI data (Raw data), preprocessed data and Excel file that support the findings of this study will be available in^89^. The data can also be requested by mail to lferrand@uji.es.
